# Novel Pharmacological Interventions to Maintain Sinus Rhythm after DC Cardioversion

**DOI:** 10.5402/2011/176834

**Published:** 2011-07-12

**Authors:** D. E. Thomas, Z. Yousef, R. A. Anderson

**Affiliations:** The Department of Cardiology, University Hospital of Wales, Heath Park, Cardiff CF14 4XN Wales, UK

## Abstract

Despite the availability of potentially curative interventions for atrial fibrillation, there remains an important role for conventional anti-arrhythmic therapy and anti-coagulation combined with direct current cardioversion. Unfortunately, the latter approach is disturbed by high recurrence rates of atrial fibrillation. In recent years, several adjunctive therapies have emerged which may facilitate the maintenance of sinus rhythm. These novel therapies and their potential mechanisms of action are reviewed in this article.

## 1. Introduction

Atrial Fibrillation (AF) is the most common sustained cardiac arrhythmia found in clinical practice [1, 2]. Its prevalence increases with age and concomitant heart disease, and it's presence is an independent predictor of mortality [3]. Once AF is initiated, changes in the electrophysiological properties of the atria occur which promote its perpetuation [4, 5]. 

Despite new insights into the pathophysiology of AF and the emergence of novel antiarrhythmic therapies, the management of this common cardiological condition remains problematic. During the past decade, a number of large randomised trials have investigated the merits of a rate versus rhythm control strategy: Pharmacological Intervention in Atrial Fibrillation (PIAF), Strategies of Treatment of Atrial Fibrillation (STAF), Rate Control Versus Electrical Conversion (RACE), Atrial Fibrillation Followup of Rhythm Management (AFFIRM), and How to Treat Chronic Atrial Fibrillation (HOT-CAFE) [6–10]. None of these studies were able to demonstrate a significant advantage of either strategy with respect to cardiovascular morbidity and mortality, whereas they all revealed an increase in hospitalisation associated with rhythm control, PIAF and AFFIRM also demonstrating a significantly higher incidence of adverse drug events amongst the rhythm control groups. In light of these observations, and given the problems of proarrhythmia and toxic end organ effects experienced with conventional antiarrhythmic medication, a consensus has emerged that rate control is at least as effective treatment option as attempting to restore SR, particularly in certain groups of patients with significant comorbidity.

There remains, however, a number of theoretical advantages to pursuing a rhythm control strategy where appropriate, and both European and American guidelines recognise this [11, 12]. The restoration of effective atrial contraction will result in improved diastolic performance, and, therefore, cardiac output [13] which translates into improved functional capacity and exercise tolerance [7, 8]. Furthermore, the restoration of sinus rhythm leads to a reversal of atrial electrical remodelling, and, hence, a reduced propensity for the development of chronic arrhythmia [14].

Electrical external cardioversion is a well-established treatment for persistent AF. Depending on patient selection, it can have very high initial success rates with studies reporting up to 95% cardioversion success [15, 16]. The problem, however, is not the efficacy of the procedure, but the very high subsequent relapse rate, even when conventional antiarrhythmic drugs are used adjunctively. This has led to growing interest and exploration of alternative pharmacotherapy, not generally thought of as directly antiarrhythmic, for the prevention of AF and the maintenance of SR after cardioversion.

This review examines the growing body of evidence that suggests that targeting the rennin angiotensin aldosterone system (RAS), reducing inflammation, and using drugs such as statins and fatty acids may offer a legitimate approach to the prevention of AF recurrence. 

## 2. The Renin Angiotensin Aldosterone System

### 2.1. ACE Inhibitors and Angiotensin II Type 1 Receptor Blockers

Angiotensin-converting enzyme inhibitors (ACEIs) and angiotensin II type 1 receptor blockers (ARBs) improve prognosis in patients with underlying cardiac disease such as chronic heart failure (CHF), ischaemic heart disease (IHD), and hypertension (HT) [17–23] irrespective of cardiac rhythm at the outset of treatment. Indeed, it was the observation of the potential ability of ACEIs to protect against the development of AF in clinical trials of CHF [24, 25] and postinfarction left ventricular (LV) dysfunction [26] that aroused interest in this area. Pedersen et al. [26], for example, in a study evaluating the role of ACEIs in patients with CHF observed that trandolapril reduced the risk of developing AF by 55%. More recently a meta-analysis has shown that in over 24,000 patients with either HT, CHF, or IHD treatment with ACEIs/ARBs markedly reduces the risk of development of AF [27]. There is also evidence to suggest that targeting RAS prevents AF relapse after electrical cardioversion. In a study by Madrid and colleagues [28], the addition of irbesartan to amiodarone achieved better maintenance of sinus rhythm than amiodarone alone during long-term followup after DCCV. Similarly, Ueng et al. [29] showed that adding enalapril to amiodarone was more effective at maintaining SR at 4 weeks than amiodarone alone (*P* = 0.002). In both studies, there was a trend towards lower rate of immediate recurrence in the combination treatment group, with most of the benefit being seen within the first 8 weeks following cardioversion

ACEIs and ARBs appear to have potentially beneficial effects on AF conversion and SR maintenance by interaction with the deleterious structural and electrical remodelling processes that can occur with AF. Three mechanisms have been suggested to explain their antiarrhythmic actions in AF:

1. reduced atrial stretch and reversal of atrial structural remodelling,

(2) suppressed angiotensin-II-induced fibrosis,

(3) modulation of ion channel function and reversal of atrial electrical remodelling.

#### 2.1.1. Reducing Atrial Stretch

Raised atrial pressure increases vulnerability to arrhythmia by shortening refractory periods, through the opening of stretch-activated ion channels. In animal studies of congestive heart failure (CHF), it has been shown that atrial stretch induces action potential shortening and delayed impulse propagation which predispose to AF [30–34]. The haemodynamic effects of ACEI include systemic arteriolar dilatation and increased large artery compliance that decrease systolic blood pressure. In the setting of CHF, the inhibition of RAS reduces afterload and systolic wall stress and decreases left atrial pressure [35].

In the study by Ueng and colleagues [29], an LA dimension of >40 mm was the only clinical parameter which predicted relapse into AF. Treatment with enalapril significantly reduced the recurrence of AF in these patients, implying reversibility of structural remodelling.

#### 2.1.2. Preventing Fibrosis

Activation of RAS leads to cardiac fibrosis in a variety of pathological conditions. Angiotensin II possesses a growth-enhancing effect on cardiac myocytes, vascular smooth muscle cells, and fibroblasts. Binding of angiotensin II to AT1 receptors stimulates fibrous tissue formation by promoting TGF-*β*1 synthesis [36]. This cytokine is activated during the development of CHF [37], and its selective overexpression shows strong correlation with an AF in animal studies [38]. Cardiac fibrosis and in particular interstitial fibrosis is commonplace in patients with AF, and the likelihood of AF increases with increasing extent of fibrosis [39]. ACEIs and ARB's have been shown to reduce atrial fibrosis, conduction abnormalities, and AF promotion while reducing atrial angiotensin II concentrations [40–42].

**The Role of Aldosterone**

Aldosterone, the final product of RAAS activation is also a potent stimulator of cardiac fibrosis [43]. When present in excess, it is associated with a greatly increased risk of atrial fibrillation [44].

Milliez and his colleagues [45] looked at the effects of spironolactone, lisinopril, and/or atenolol on atrial arrhythmias, haemodynamics, and atrial fibrosis in rats with CHF. They found that each treatment and combination suppressed atrial ectopy in a statistically significant fashion, but only spironolactone alone or in combination significantly attenuated the arrhythmogenic process of atrial fibrosis.

Aldosterone blockade may be a promising new approach for atrial tachyarrhythmia prevention in CHF. Selective aldosterone-receptor blockade with eplerenone suppressed inducible AF in an experimental model of heart failure [46]. In this study, eplerenone treatment was associated with selective prolongation of atrial refractory periods and improved LV diastolic filling

The placebo-controlled EPLERAF study which is currently underway will examine whether Eplerenone reduces atrial fibrillation (AF) recurrences within the first 8 weeks after electrical cardioversion of persistent AF.

#### 2.1.3. Modulating Cellular Electrophysiology and Electrical Remodelling

AF recurrence after DC cardioversion (DCCV) can be the clinical consequence of electrical remodelling through, for example, the effects of atrial premature beats and the shortening of effective refractory periods within the atria [4, 47]. Atrial premature beats (APBs) are a potentially important trigger for the induction and reinitiation of AF [48]. The ability of APBs to induce AF depends on their timing and location relative to electrical heterogeneity gradients [49]. ACEIs and ARBs may suppress the APBs, by modifying the sympathetic tone [50, 51] and altering Na^+^/Ca^2+^ exchanger activity [52–54]. They may also attenuate effective refractory period shortening.

A recent study by Zaman et al. [55] showed that patients listed for routine DCCV who were simultaneously taking ACEIs had a decreased number of defibrillation attempts and decreased AF-related admission rates. Furthermore, the investigators identified a decrease in signal averaged P-wave duration (prolonged P-wave duration is a powerful marker of resistant AF). Therefore, in this study, ACEIs facilitated AF cardioversion and the maintenance of SR by modifying the arrhythmogenic substrate.

Schneider et al. recently published a meta-analysis of 8 RCT's which investigated the effects of RAS inhibition on the recurrence of AF after electrical or chemical cardioversion (Table 1). Overall, they report a significant reduction in the risk for AF recurrence after cardioversion with the use of ACEI's or ARBs (OR: 0.55; 95% CI: 0.34 to 0.89; *P* = 0.01). However, 4 of these trials were open-label design [27–29, 56], and their promising results were not replicated in the double-blind, placebo-controlled “candesartan in the prevention of relapsing atrial fibrillation” (CAPRAF) study [57], or in the GISSI-AF trial [58], which failed to detect any effect of treatment with valsartan on the recurrence of AF. 

Further work, therefore, needs to be done in this area to clearly define the therapeutic role of RAS blockade in AF. Additional studies will also need to ascertain whether upstream blockade of the RAA axis with ACEI is superior to downstream blockade with ARBs, or whether there is additional advantage in combining treatments.

## 3. The Role of Inflammation in AF

Inflammation plays a pivotal role in the pathogenesis of coronary atherosclerosis and cardiovascular disease [61], and markers of inflammation such as C-reactive protein (CRP) have been shown to be good predictors of future cardiovascular events [62–64]. There is growing interest in both the role that inflammation has in the propagation of persistent AF and also the role of inflammatory indices to predict successful cardioversion and maintenance of SR long term. 

Bruins and colleagues were the first group to propose the inflammation—AF hypothesis, following their observations of an increased frequency of AF after coronary artery bypass surgery [65]. They chronologically paralleled changes in the inflammatory cascade resulting in complementary activation with rising incidence of atrial arrhythmias. 

Since their formative work, there have been numerous clinical studies demonstrating the relationship between inflammation (using serum or plasma vascular inflammatory markers) and AF [66–72]. Chung and colleagues showed that amongst their cohort of 131 patients with atrial arrhythmias, those with persistent AF had higher CRP levels than those with paroxysmal AF, who in turn had higher levels than normal controls [72]. 

In certain subgroups of patients, AF may primarily be an inflammatory disorder, in which inflammatory change acts as both the initiator, and the propagator of AF. The presence of inflammatory infiltrates, and oxidative damage in the atrial biopsy specimens of patients with lone AF is well documented [39]. In a study by Nakamura and colleagues [73], abnormal atrial histology was uniformly found in multiple biopsy specimens of patients with lone AF compared with normal histology in all of the controls, with 66% of the AF group showing evidence of occult myocarditis.

## 4. Corticosteroids

Given the evidence supporting the link between inflammation and AF, it is logical to question whether drugs with an anti-inflammatory action have a therapeutic and preventative role in certain patient subsets. Dernellis and Panaretou showed that the addition of low-dose glucocorticoid therapy to established antiarrhythmic treatment after cardioversion produced a dramatic reduction in AF recurrence rates and the development of permanent AF. Their study also confirmed the predictive value of CRP measurement in such circumstances, showing that the risk of recurrent AF was increased by approximately 7 times for each 1 mg/dL increase in plasma levels of CRP [74].

Whilst the current evidence base to advocate the use of steroids as routine treatment for AF is absent, there appears to be future scope for their use in maintaining sinus rhythm following DCCV, particularly amongst those with high risk of recurrence as indicated by elevated inflammatory markers.

## 5. Statins

Statins (hydroxymethylglutaryl coenzyme A reductase inhibitors) have anti-inflammatory actions independent of their lipid lowering effect and have been shown to lower CRP [62, 75, 76]. In a retrospective study carried out by Siu et al., they looked at 62 patients over 18 months who underwent DCCV for lone persistent AF. At the end of a mean patient followup of 44 months, they discovered a high overall recurrence rate of 85%, but a significant difference between those taking and not taking statin therapy (40% versus 84%, *P* = 0.007). The relative benefit of statin therapy was observed during the first few months after DCCV and persisted throughout followup [77].

A number of different mechanisms by which statins help prevent AF recurrence, in addition to their anti-inflammatory role, can be postulated.

Ischaemia is a known substrate for AF [78], and, therefore, the beneficial effects they have in this setting may be due to their retardation of coronary artery disease progression. It is also possible that statins exert direct antiarrhythmic effects by altering the fatty acid composition of cell membranes which has a knock-on effect on the function of transmembrane ion channels [79, 80].

## 6. Polyunsaturated Fatty Acids

*N*-3 polyunsaturated fatty acids (PUFAs), of which oily fish are an important source, are believed to possess both anti-inflammatory and antiarrhythmic effects and, thus, may have a role in the prevention and treatment of AF [81]. PUFA's have been shown to exert anti-asynchronous effects in rat atrial myocytes, reduce proarrhythmic eicosanoids, and inhibit sodium and calcium currents which contribute to the arrhythmic process [82, 83]. *N*-3 fatty acids have also been shown to be associated with a greatly reduced risk of sudden cardiac death [84, 85].

Only a small number of randomised controlled studies have been published on the therapeutic use of fish oils in AF. In a recent study examining the role of *N*-3 fatty acids in preventing AF following coronary bypass surgery; it showed that preoperative use of PUFA's (2 g for 5 days prior to surgery and continued until discharge) was associated with a 54% relative risk reduction of postoperative AF [86]. This is comparable to rates achieved with conventional antiarrhythmic therapy. To date, no one has looked at whether this benefit is replicated peri-DCCV.

In a prospective, population-based cohort of over 65 year olds, Mozaffarian and colleagues were able to demonstrate that the consumption of high levels of fish containing omega-3 fatty acids was associated with a significantly lower incidence of subsequent AF development. However, these results were challenged by a much larger prospective cohort study that failed to demonstrate any reduction in risk of AF or flutter associated with high consumption of omega-3 fatty acids. What is clearly now needed is a randomised-controlled study examining the potential role of PUFA's in preventing recurrence of AF following successful DCCV.

## 7. Summary

In certain subgroups of patients with AF, successful maintenance of SR has the potential to offer significant advantage and improved clinical outcome when compared to rate control strategies. The long-term success of DC cardioversion, however, remains poor. In this review, we have shown that there are additive pleiotropic effects of many medications that patients already commonly take for coexisting conditions associated with AF. When considering cardioverting patients with AF, these concomitant therapies may potentially facilitate SR maintenance and, therefore, allow tailoring of drug treatment in individual patients depending on the underlying aetiology of their arrhythmia. 

AF remains a condition for which there is still significant unmet need. Increased use of ablative therapy will help to bridge future treatment gaps, as well as the use of upstream therapies and novel pharmacotherapy peri-cardioversion.

## Figures and Tables

**Table 1:** 

Study	Study design	*N*	Followup (months)	ACE/ARB	Comparator drug	Main findings
Van den Berg et al. [59]	Randomised, double blind	18	1.5	Lisinopril	Placebo	Maintenance of SR: 71% with lisinopril versus 36% with placebo

Madrid et al. [28]	Randomised, open label	154	2	Irbesartan	No irbesartan	Reduction in the risk of AF recurrence with irbesartan: adjusted HR (95% CI): 0.19 (0.04–0.86); *P* = 0.03

Ueng et al. [29]	Randomised, open label	145	9	Enalapril	No enalapril	Recurrence of AF: 4.3% with enalapril versus 14.7% without, *P* = 0.067. Probability of remaining in SR at 4 weeks: 84.3 versus 61.3%, *P* = 0.002. Adjusted HR (95% CI): 0.37 (0.12–1.15); *P* = 0.041

Madrid et al. [27]	Randomised, open label	60	7.3	Irbesartan (+amiodarone)	No irbesartan	Patients remaining free of AF at 1 yr: irbesartan 300 mg + amiodarone = 77%, irbesartan 150 mg + amiodarone = 65%, amiodarone alone = 52% (*P* = 0.001 versus irbesartan 300 mg + amiodarone)

Grecu et al. [56]	Randomised, open label	36	12	Perindopril (+Propafenone)	Placebo	Maintenance of SR: 37% with perindopril versus 20% with placebo. Time interval to recurrences was significantly higher in ACEI-treated patients (7.06 ± 1.02 versus 4.50 ± 0.93 months; *P* = 0.034)

Tveit et al. [57] (CAPRAF study)	Randomised, double blind	137	6	Candesartan	Placebo	Recurrence of AF: 71% with candesartan versus 65% with placebo *P* = ns

Belluzzi et al. [60]	Randomised, double blind	62	36	Ramipril	Placebo	Recurrence of AF: 10% with ramipril versus 32% with placebo (*P* < 0.03, Kaplan-Meier, log-rank test)

Disertori et al. [58] (GISSI-AF)	Randomised, double blind	1,442	12	Valsartan	Placebo	Recurrence of AF: 51.4% with valsartan versus 52.1% with placebo. Adjusted HR (96% CI): 0.97 (0.83–1.14); *P* = 0.73
